# Transthyretin-Related Cardiac Amyloidosis: A Case of Delayed Diagnosis in the Comorbid Patient and Literature Review

**DOI:** 10.7759/cureus.89851

**Published:** 2025-08-11

**Authors:** Akshaye Patel, Immy Stringer, Leyan Edhem, Gedoni Eni, Rebecca Delamere, Adnan Ahmed, Jhiamluka Solano

**Affiliations:** 1 Internal Medicine, Northern Lincolnshire and Goole NHS Foundation Trust, Scunthorpe, GBR; 2 General Internal Medicine, Scunthorpe General Hospital, Scunthorpe, GBR; 3 Cardiology, Scunthorpe General Hospital, Scunthorpe, GBR; 4 Internal Medicine, Scunthorpe General Hospital, Scunthorpe, GBR; 5 Cardiology, Hull University Hospital (Castle Hill Hospital), Hull, GBR; 6 Resident Doctor Committee, Royal College of Physicians, London, GBR; 7 Education Committee, Academy of Medical Educators, Cardiff, GBR

**Keywords:** 99mtc-dpd scintigraphy, cardiac amyloidosis, conduction abnormalities, heart failure with preserved ejection fraction (hfpef), transthyretin (attrwt)

## Abstract

We report the case of an 82-year-old male with a history of bronchiectasis, asthma, and atrial fibrillation, who presented with progressive exertional dyspnoea, peripheral oedema, and recurrent heart failure exacerbations. Initial management targeted presumed pulmonary decompensation. Elevated natriuretic peptides, echocardiographic evidence of concentric left ventricular hypertrophy with preserved ejection fraction, and progressive conduction abnormalities prompted further evaluation. A 99mTc-DPD scintigraphy scan revealed Perugini grade 3 myocardial uptake consistent with wild-type transthyretin (ATTRwt) cardiac amyloidosis. Serum and urine studies excluded light-chain amyloidosis. Despite the presence of classical ‘red flag’ features, including atrial fibrillation, carpal tunnel syndrome, and unexplained left ventricular hypertrophy, diagnosis was significantly delayed by approximately 18 months, from initial symptom onset to definitive diagnosis, during which recurrent hospitalisations and progressive functional decline occurred. Earlier features were overlooked due to the attribution of symptoms to coexistent pulmonary disease and chronic kidney dysfunction. This case highlights the diagnostic challenges posed by ATTRwt, particularly in multimorbid older adults. Overlapping features with respiratory and renal pathology, as well as age-associated cardiovascular changes, obscure the clinical picture. Awareness of hallmark extracardiac features, systematic use of cardiac imaging, and prompt nuclear scintigraphy are essential for timely diagnosis. Early identification may enable consideration of disease-modifying therapy and improved symptom management.

## Introduction

Heart failure with preserved ejection fraction (HFpEF) accounts for approximately half of all heart failure cases and is increasingly recognised as a heterogeneous clinical syndrome with diverse underlying aetiologies. Among these, transthyretin-related cardiac amyloidosis (ATTR-CA) is an important and often underdiagnosed cause, particularly in older adults with unexplained HFpEF, atrial arrhythmias, and increased left ventricular wall thickness without a history of hypertension or aortic stenosis [1].

ATTR-CA results from the deposition of misfolded transthyretin proteins in the myocardial extracellular matrix, leading to progressive diastolic dysfunction, conduction abnormalities, and heart failure symptoms. It exists in two forms: hereditary (ATTRv), associated with TTR gene mutations, and wild-type (ATTRwt), previously known as senile systemic amyloidosis, which occurs in elderly individuals without a genetic predisposition [2]. ATTRwt has a male predominance and is often associated with comorbid conditions such as carpal tunnel syndrome, lumbar spinal stenosis, and atrial fibrillation, frequently leading to delayed or missed diagnosis [3]. Epidemiological studies suggest that ATTRwt may be present in up to 13-16% of HFpEF patients with increased wall thickness, yet median diagnostic delays of one to three years are common and are associated with progressive functional decline, worsening heart failure, and reduced survival [4]. We present a case of wild-type ATTR cardiac amyloidosis in an elderly male with coexisting pulmonary disease and recurrent decompensated heart failure, in whom the diagnosis was delayed due to overlapping clinical features.

## Case presentation

An 82-year-old male initially presented to his general practitioner with progressive exertional dyspnoea, bilateral ankle oedema, and a chronic productive cough with white sputum. He was empirically treated for an asthma exacerbation with inhalers. At subsequent review, he appeared clinically fluid overloaded, and he was commenced on a trial of spironolactone, and routine investigations were requested, including BNP. The BNP level was noted to be significantly elevated at over 4000. Despite the initial community interventions while awaiting secondary care review, his symptoms continued to worsen, and he became increasingly immobile, prompting a referral to acute care services.

His past medical history included hypertension, asthma, bronchiectasis, atrial fibrillation, chronic kidney disease stage 3b without proteinuria, carpal tunnel syndrome, and prior carcinoma of the glottis and larynx treated with radiotherapy. He is an ex-smoker and has a history of superficial basal cell carcinoma, solar keratosis, cervical spondylosis, osteoarthritis with previous right knee replacement, umbilical hernia repair, and gastroesophageal reflux disease.

**Figure 1:**
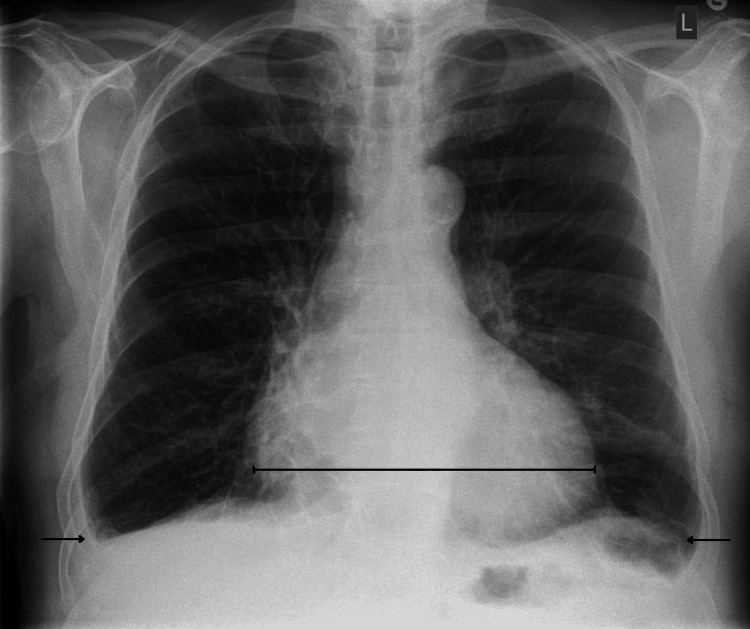
Chest X-ray Cardiomegaly (bracket line) with bilateral pleural effusions (arrows).

**Figure 2:**
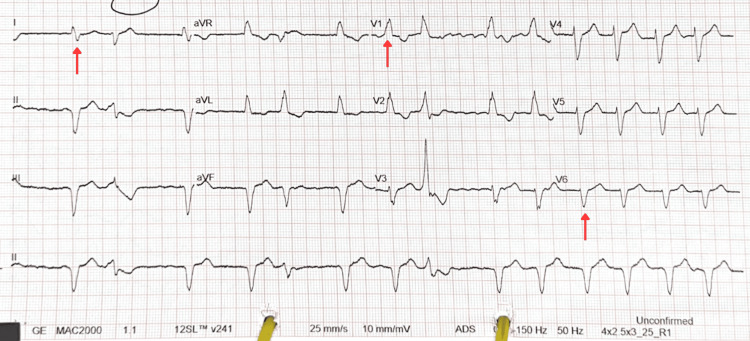
Twelve-lead ECG The ECG demonstrates atrial fibrillation, evidenced by an irregular R-R interval with no distinct P waves. Additionally, a right bundle branch block is present, as indicated by a widened QRS complex with an rsR' pattern in lead V1 (arrow) and broad terminal S waves in leads I and V6 (arrows).

On evaluation in the same-day emergency care unit, chest radiography revealed cardiomegaly with bilateral pleural effusions (Figure 1). Electrocardiography demonstrated atrial fibrillation with right bundle branch block and occasional ventricular ectopics with a right bundle morphology reflecting a left ventricle origin (Figure 2). Clinically, he exhibited bilateral lower limb oedema extending to the knees, with no raised jugular venous pressure and clear chest auscultation. He was commenced on oral furosemide with improvement noted both subjectively and on clinical reassessment. An outpatient transthoracic echocardiogram and cardiology follow-up were arranged. Baseline blood tests showed no significant changes compared to previous recorded results (Table 1).

**Table 1:** Summary of initial laboratory investigations with reference ranges ALT - alanine aminotransferase; ALP - alkaline phosphatase; eGFR - estimated glomerular filtration rate; GGT - gamma-glutamyl transferase; Hb - haemoglobin; PLT - platelets; TSH - thyroid-stimulating hormone; WCC - white cell count

Investigation	Patient’s results	Reference range
eGFR	33	>90 mL/minute/1.73 m²
Creatinine	166	60-110 µmol/L
WCC	10.1	4.0-11.0 × 10⁹/L
Hb	135	130-170 g/L (male), 120-150 g/L (female)
PLT	121	150-400 × 10⁹/L
TSH	3.3	0.4-4.0 mIU/L
ALT	59	7-56 U/L
ALP	83	40-130 U/L
GGT	236	10-71 U/L

**Figure 3:**
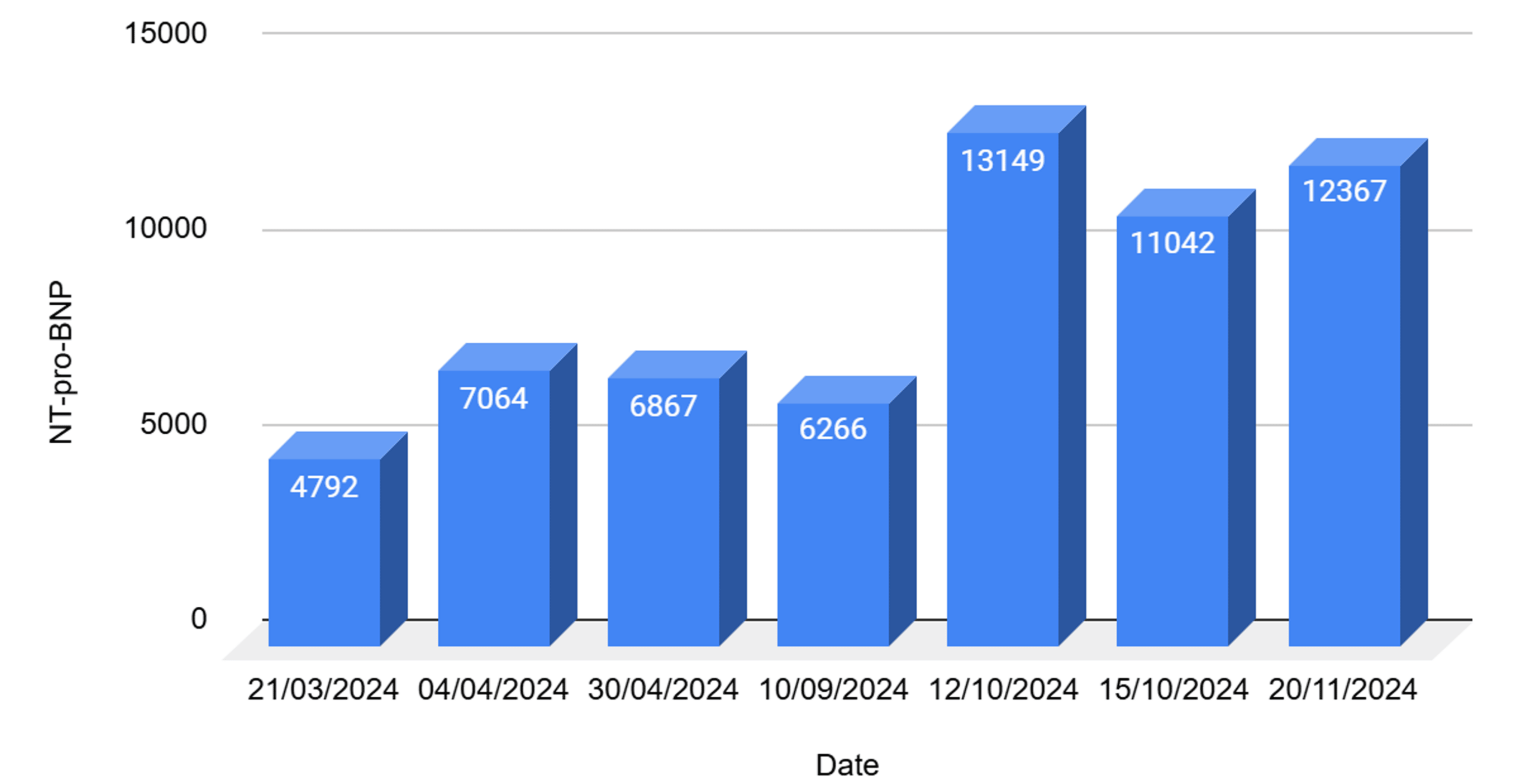
BNP trends during inpatient admissions BNP- B-type natriuretic peptide; NT-pro-BNP - N-terminal pro-B-type natriuretic peptide

Echocardiography revealed severe concentric left ventricular hypertrophy with preserved systolic function (left ventricle ejection fraction (LVEF) 74%), bi-atrial enlargement, abnormal right ventricular longitudinal function with a tricuspid annular plane systolic excursion (TAPSE) of 14 mm, and mild mitral and tricuspid regurgitation. These findings were consistent with HFpEF. On cardiology review, examination findings included raised JVP, mild unilateral leg oedema, and reduced air entry with wheeze. BNP had doubled, indicating ongoing decompensation (Figure 3). The patient was classified as New York Heart Association (NYHA) I, and heart failure pharmacotherapy was initiated and subsequently optimised, including valsartan, bisoprolol, spironolactone, furosemide, and dapagliflozin.

**Figure 4:**
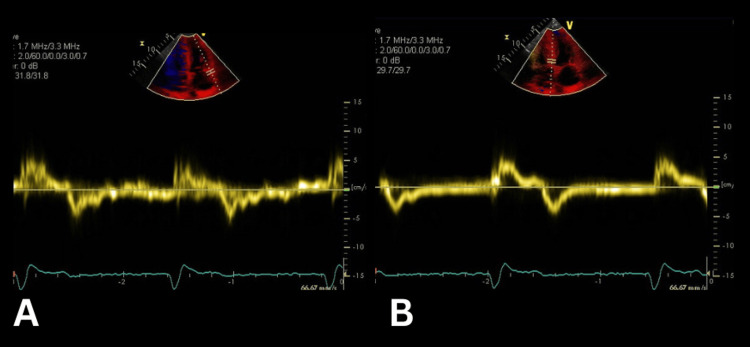
Tissue Doppler imaging (TDI) during transthoracic echocardiogram (TTE) (A) Lateral S' (mitral annulus) mean velocity of 4.7 m/second (normal >5 m/second). (B) Septal peak E' velocity of 5.1 m/second (normal >6 m/second).

Several months later, the patient was admitted to the hospital with dizziness and worsening dyspnoea. ECG at that time showed atrial fibrillation with complete heart block and a bradycardic rhythm of 41 bpm. BNP was markedly elevated, and he experienced an inpatient fall while anticoagulated with edoxaban. CT brain showed no acute haemorrhage or ischaemia. A permanent single-chamber VVI pacemaker was inserted, which was later confirmed to be functioning appropriately. A repeat echocardiogram revealed persistent findings of severe concentric LVH and bi-atrial enlargement, with reduced mitral annular longitudinal velocity (Video 1). Additionally, reduced longitudinal function was noted (mitral annulus mean velocity of 4.7 m/second and septal peak E' velocity of 5.1 m/second) (Figure 4). 

**Video 1:** Transthoracic echocardiogram on admission (A, B) Parasternal long-axis view. (C) Parasternal short-axis view. (D) Apical five-chamber view.

**Figure 5:**
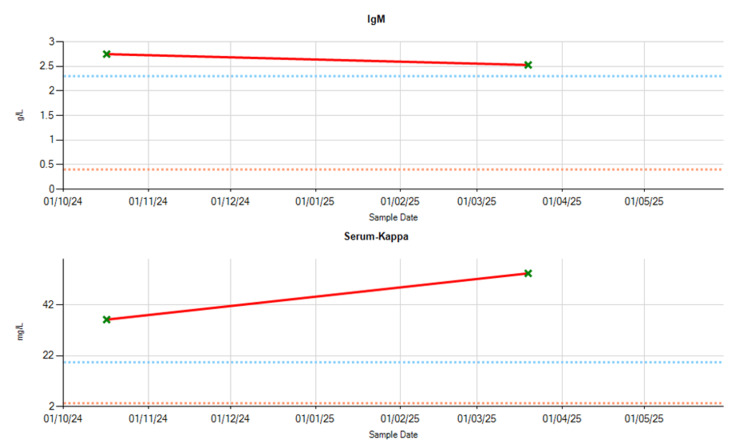
Serum IgM and serum kappa levels

**Figure 6:**
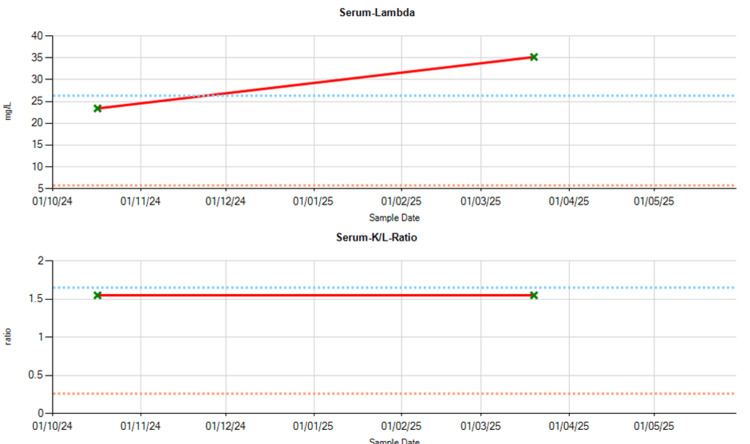
Kappa-lambda ratio blood test results

**Figure 7:**
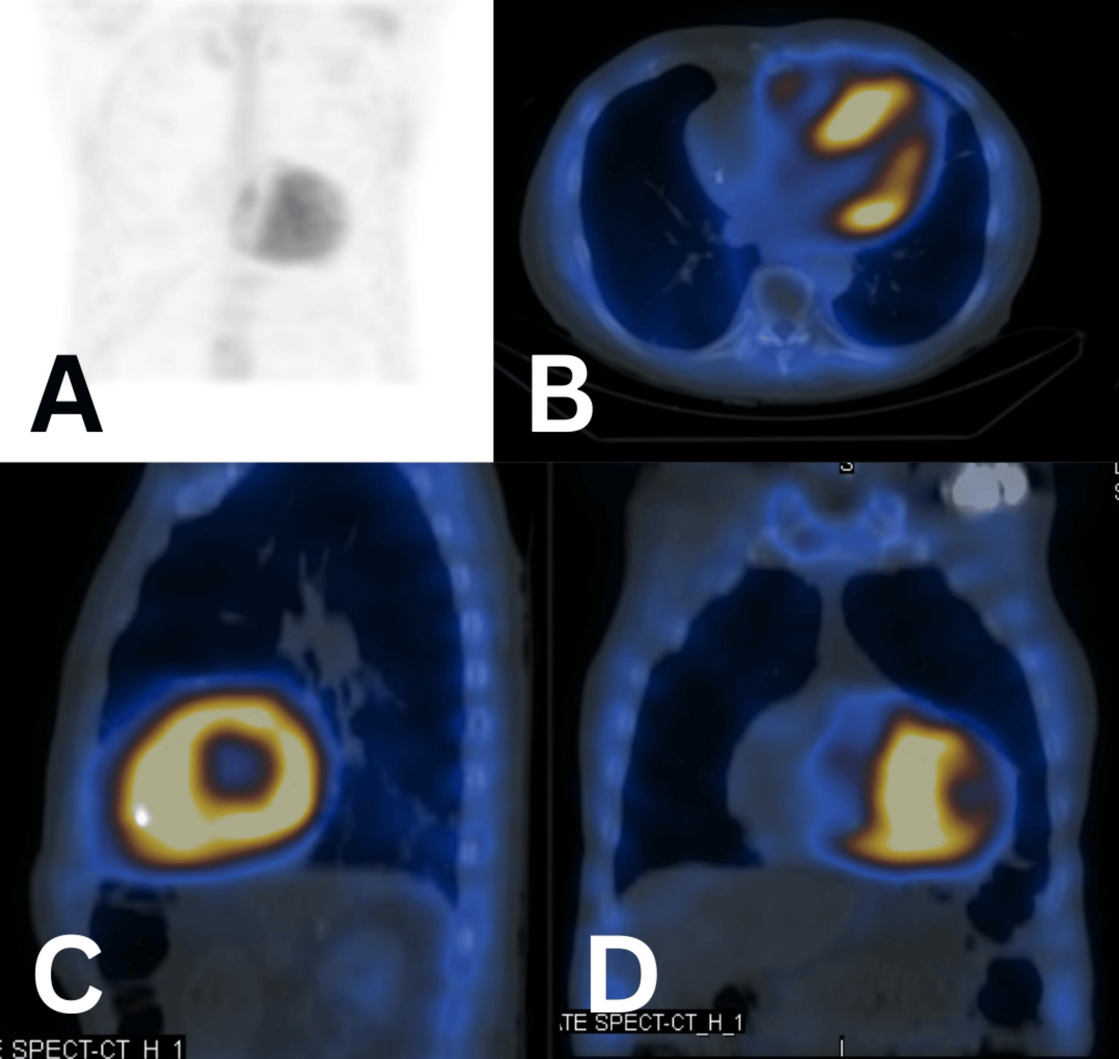
99mTc-DPD nuclear scan (A) Preferential bone tracer view. (B) Transverse view. (C) Sagittal view. (D) Coronal view.

Due to increasing concerning features, cardiac amyloidosis was considered as part of the differential diagnosis, and haematological evaluation was undertaken. Serum studies revealed elevated IgM levels (Figure 5) and abnormal kappa and lambda light chain levels, with a mildly abnormal kappa-lambda ratio (Figure 6). The patient’s functional status had declined to NYHA II, with worsening exertional dyspnoea, orthopnoea, peripheral oedema, and bibasal crackles on auscultation. A 99mTc-DPD nuclear scan was arranged to evaluate for transthyretin-related (ATTR) cardiac amyloidosis (Figure 7). The 99mTc-DPD scan revealed diffuse myocardial tracer uptake with apical sparing and involvement of both ventricles and atria, consistent with Perugini grade 3 ATTR cardiac amyloidosis. Repeat serum light chain analysis was within normal limits, and there was no monoclonal protein on serum electrophoresis or immunoglobulin studies, effectively ruling out amyloid light (AL) chain amyloidosis. The diagnosis of wild-type ATTR cardiac amyloidosis was established.

Shortly after, he was readmitted with worsening breathlessness and a productive cough. Sputum cultures repeatedly isolated Haemophilus influenzae, and despite multiple courses of doxycycline, symptoms persisted. Respiratory evaluation led to a diagnosis of bronchiectasis and asthma, supported by raised total IgE and sensitisation to house dust mite. Inhaler therapy was revised, a flutter device was introduced, and a referral was made to pulmonary rehabilitation.

Following this, the patient continued to experience recurrent episodes of fluid overload. Diuretic therapy was frequently adjusted by the cardiac nurse specialist team based on daily weights and symptoms. His NYHA classification fluctuated between II and III. Given the confirmed diagnosis of cardiac ATTR, a referral was made to the National Amyloidosis Centre to assess eligibility for Tafamidis therapy.

Serial investigations over the following months, including multiple chest radiographs and echocardiograms, documented stable cardiomegaly, persistent hyperinflated lung fields, and imaging changes consistent with chronic pulmonary and cardiac pathology. Echocardiographic findings remained consistent, with preserved left ventricular systolic function, severe concentric hypertrophy, bi-atrial dilatation, and mild valvular regurgitation. Pacemaker follow-up has confirmed satisfactory device function and longevity. The patient continues to be monitored in cardiology, respiratory, and pacing clinics and awaits specialist review for consideration of disease-modifying therapy with Tafamidis.

## Discussion

Transthyretin amyloid cardiomyopathy (ATTR-CM) exemplifies a complex disorder often with clinical presentations that make timely recognition difficult. Frequently described as rare, the actual prevalence is unknown; however, an autopsy study suggests a potential prevalence of around 25% [5]. An American study using a claims database [6] suggested an increase in prevalence from 20.1 to 50.1 cases per million over a nine-year period (2007-2015). In contrast, a single-centre Spanish study revealed that 13% of its HFpEF patients had ATTR-CMwt [4]. 

Most patients initially report non-specific symptoms like progressive exertional dyspnoea, fatigue, and ankle oedema, typical of HFpEF [7], often attributed to other causes, especially given the commonly affected elderly age group. This was the case for our patient, who had both asthma and bronchiectasis and was initially treated as a presumed exacerbation of these conditions. This delayed investigation for alternative causes of the patient’s symptoms, such as ATTR-CM. Additionally, when reviewing heart failure symptoms as a result of ATTR-CM, such as peripheral oedema and raised BNP, it is important to consider concurrent comorbidities that may present with similar symptoms and signs. For example, this patient had CKD, which can independently cause elevated BNP levels and fluid retention, potentially complicating the interpretation of the result and, thus, delaying the diagnosis of ATTR-CM [8].

Conduction disease, atrial fibrillation or complete heart block may dominate. Classic amyloid clues such as discordant QRS voltage on ECG and wall thickness on echocardiography are absent in many cases [7]. These protean features overlap common geriatric phenotypes like hypertensive left-ventricular hypertrophy, hypertrophic cardiomyopathy and aortic stenosis, explaining why more than 40 % of patients remain mislabelled for around four years after symptom onset [9]. Nonetheless, preventing misdiagnosis demands systematic vigilance, especially when features like vanishing hypertension, low-flow, low-gradient severe aortic stenosis, as well as extracardiac findings including bilateral carpal-tunnel syndrome, lumbar spinal-stenosis, arthropathy or polyneuropathy are present. These features often precede cardiac involvement by years and should prompt a raised index of suspicion for ATTR-CM in the appropriate context [4,7,10]. In our case, the patient had a history of carpal tunnel syndrome, which may have been caused by amyloid deposition.

Diagnosis of cardiac amyloidosis relies on sequential investigation, starting with electrocardiography and cardiac imaging. Characteristic ECG changes include low-voltage QRS complexes, conduction abnormalities, and bundle branch block, as well as atrial fibrillation [11]. The difficulty lies in identifying these changes as specific in a comorbid population in which arrhythmias become more common, and low voltage can be caused by obesity, hypothyroidism and chronic obstructive lung disease instead, as showcased in our patient. These changes have been reported to occur in a later phase of cardiac amyloidosis development and in a variable pattern [12].

Echocardiography also plays a crucial role in assessing both the systolic and diastolic capacity of the heart, as well as valvular function simultaneously. This is especially important, as various studies support that 10-20% of those with HFpEF eventually receive an ATTR-CA diagnosis [13,4]. As a form of restrictive cardiomyopathy caused by the deposition of amyloid fibrils and collagen, which affects myofibril function, as well as direct and preceding myocyte toxicity from the same oligomers [14], cardiac amyloidosis typically presents with an ejection fraction greater than 50% in the majority of patients, as in this case. A high burden of amyloid involvement can, however, impair systolic function in some patients with late-stage disease [11,15]. 

Left ventricular hypertrophy is a common finding, particularly concentric thickening, which is more pronounced in ATTR-CA than in other forms of HFpEF [4]. The involvement of right-sided hypertrophy is more suggestive of the condition. Atrial dilatation and valve thickening are often seen [11]. A refractile and granular myocardium, as well as longitudinal contraction (rather than radial contraction) impairment, the latter reported in our study, are also findings suggestive of the condition and often more significant in the context of a preserved ejection fraction [15]. As described, our patient exhibited many of these findings; however, it can be challenging to attribute them directly to ATTR-CA, and they may not be observable with standard two-dimensional echocardiography. Additional investigations will always be required to confirm the diagnosis.

The problem with the use of echocardiography diagnosis lies in its limited specificity (similarly to ECG results) in the absence of other symptoms, where findings of hypertrophy may be thought due to hypertension-induced remodelling or concurrent pre-existing valve disease, and arrhythmia and HFpEF assigned to age in a condition that often affects patients, and males, in their seventh and eighth decades. From a holistic assessment, a record of peripheral or autonomic nerve involvement, which often precedes cardiac involvement and was present in this patient’s history [16], would also increase the likelihood of a diagnosis of ATTR-CA.

Although cardiac MRI can detect gadolinium accumulation in areas of amyloid deposits, technetium scintigraphy is the most commonly used advanced imaging technique for diagnosis. Traditionally, histological diagnosis was required, along with Congo red staining; however, endomyocardial biopsy carried the risk of tamponade and infarction due to technical difficulties, while many studies report a low yield of results for ATTR-CA from abdominal fat sampling. This non-invasive method often reveals diffuse tracer uptake, with ventricles, atria and valves affected. It is believed to have >95% sensitivity and >85% specificity for diagnosis of ATTR-CA, with the false positives obtained from AL amyloidosis removed with assessment of serum or urine light chains; the positive predictive value becomes 100% in evidence of myocardial tracer uptake and absence of monoclonal bands [16,3], supporting the diagnosis in our patient following the repeat light chain panel. It is thought that technetium uptake occurs before characteristic echocardiographic findings manifest; however, cadaveric studies have shown that non-specific echocardiographic findings suggestive of the condition may present first and in a greater number of patients, with uptake on nuclear imaging following once there is a significant burden of amyloid deposition in the myocardium [17,18].

Arrhythmias and conduction system disease are hallmark features of ATTR-CA [19]. At the time of diagnosis, approximately 9-10% of patients already have high-grade atrioventricular (AV) block requiring permanent pacemaker implantation. During follow-up, an additional 10-12% may develop advanced conduction disease necessitating pacing, with higher rates observed in individuals with wild-type ATTR-CA (wtATTR-CA) [20,4]. Overall, pacemaker implantation is required in approximately 20-23% of patients over a median follow-up of 2.5 to three years, with a higher incidence in wtATTR-CA due to the progressive involvement of the His-Purkinje system [21,22]. Key predictors include prolonged QRS duration, a PR interval greater than 200 ms, and the presence of AF. 

Regarding treatment, the ATTR-ACT trial was conducted to evaluate the efficacy of Tafamidis in patients with ATTR-CA. Transthyretin is a protein that is synthesised in the liver and transports thyroxine and retinol-binding protein-retinol (vitamin A complex). Tafamidis binds to the thyroxine-binding sites of transthyretin with high affinity, selectively inhibiting tetramer dissociation and amyloidogenesis. It is the first therapy approved to slow the progression of peripheral neurologic impairment in transthyretin amyloid polyneuropathy [19]. It is advised that patients presenting with preserved functional status yet a high symptom burden should be considered for a diagnosis of ATTR-CA [22]. Our patient initially had preserved functional status (NYHA class I); however, he had advanced imaging findings. Also, his symptoms got progressively worse despite treatment. Such discordance between objective imaging and subjective symptom burden is characteristic of early-stage ATTR-CA [19]. It emphasises the importance of maintaining a high index of clinical suspicion in patients with initially preserved functional status but who develop multiple, progressive symptoms and clinically deteriorate despite treatment.

## Conclusions

This case illustrates the importance of maintaining a high index of suspicion for cardiac amyloidosis, particularly ATTRwt, in elderly patients presenting with unexplained HFpEF, conduction abnormalities, and suggestive extracardiac features such as carpal tunnel syndrome. In the presence of overlapping comorbidities, diagnostic delays are common and may impact therapeutic options. In this patient, the diagnosis was delayed by approximately 18 months from initial symptom onset, during which functional status progressively declined. Earlier detection could have facilitated the timely initiation of disease-specific therapy, potentially improving prognosis and quality of life. Tafamidis remains the only approved disease-modifying treatment for ATTR-CM, underscoring the urgency of early recognition. Long-term follow-up with a multidisciplinary team, including cardiology, neurology, and supportive care, is essential to optimise outcomes and address disease progression. A structured, multimodal diagnostic approach, encompassing clinical vigilance, echocardiographic assessment, serum light-chain analysis, and nuclear scintigraphy, is crucial for enabling an accurate diagnosis and timely referral for consideration of disease-specific treatments, such as Tafamidis.
